# SARS-CoV-2 Spike-Binding Antibody Longevity and Protection from Reinfection with Antigenically Similar SARS-CoV-2 Variants

**DOI:** 10.1128/mbio.01784-22

**Published:** 2022-08-23

**Authors:** John Kubale, Charles Gleason, Juan Manuel Carreño, Komal Srivastava, Gagandeep Singh, PARIS Study Team, Aubree Gordon, Florian Krammer, Viviana Simon

**Affiliations:** a Department of Epidemiology, School of Public Health, University of Michigan, Ann Arbor, Michigan, USA; b Department of Microbiology, Icahn School of Medicine at Mount Sinai, New York, New York, USA; c Department of Medicine, Division of Infectious Diseases, Icahn School of Medicine at Mount Sinai, New York, New York, USA; d Global Health and Emerging Pathogens Institute, Icahn School of Medicine at Mount Sinai, New York, New York, USA; e Department of Pathology, Molecular and Cell Based Medicine, Icahn School of Medicine at Mount Sinai, New York, New York, USA; f Center for Vaccine Research and Pandemic Preparedness, Icahn School of Medicine at Mount Sinai, New York, New York, USA; McMaster University

**Keywords:** SARS-CoV-2, COVID-19, spike-binding antibodies, protection, modeling, antibody durability

## Abstract

The PARIS (Protection Associated with Rapid Immunity to SARS-CoV-2) cohort follows health care workers with and without documented coronavirus disease 2019 (COVID-19) since April 2020. We report our findings regarding severe acute respiratory syndrome coronavirus 2 (SARS-CoV-2) spike-binding antibody stability and protection from infection in the pre-variant era. We analyzed data from 400 health care workers (150 seropositive and 250 seronegative at enrollment) for a median of 84 days. The SARS-CoV-2 spike-binding antibody titers were highly variable with antibody levels decreasing over the first 3 months, followed by a relative stabilization. We found that both more advanced age (>40 years) and female sex were associated with higher antibody levels (1.6-fold and 1.4-fold increases, respectively). Only six percent of the initially seropositive participants “seroreverted.” We documented a total of 11 new SARS-CoV-2 infections (10 naive participants and 1 previously infected participant without detectable antibodies; *P* < 0.01), indicating that spike antibodies limit the risk of reinfection. These observations, however, only apply to SARS-CoV-2 variants antigenically similar to the ancestral SARS-CoV-2 ones. In conclusion, SARS-CoV-2 antibody titers mounted upon infection are stable over several months and provide protection from infection with antigenically similar viruses.

## INTRODUCTION

Severe acute respiratory syndrome coronavirus 2 (SARS-CoV-2) caused the coronavirus disease 2019 (COVID-19) pandemic with over 430 million infections (WHO dashboard, 25 February 2022) since it emerged in late 2019 (1, 2). In the vast majority of individuals, infection with SARS-CoV-2 leads to the induction of a specific adaptive immune response, including spike-binding as well as neutralizing antibodies (3, 4). Indeed, we found that over 90% of individuals infected during the first wave in New York City (NYC) had robust antibody titers as measured using an enzyme-linked immunosorbent assay (ELISA) (>30,000 cross-sectional measurements) (4, 5). The durability and protective effect of such antibody responses remains a topic of active investigation even as we move into the third year of the pandemic. An initial study (6) reported fast waning of SARS-CoV-2 binding antibodies, but others report spike-binding IgG antibodies being detectable months after infection (7–9).

The first SARS-CoV-2 infection in New York State was officially detected at the Mount Sinai Health System in NYC on 29 February 2020, although SARS-CoV-2 had likely been introduced to the local communities weeks to months earlier (10, 11). Indeed, the New York metropolitan area emerged as one of the early COVID-19 epicenters in the United States. This initial COVID-19 wave was exponential in growth and nearly overwhelmed our local health care systems due to the high number of patients with severe COVID-19 manifestations, resulting in infection fatality rates ranging between 1% to 1.5% (11, 12). It was at that point in time (April 2020) that we started enrollment for the PARIS (Protection Associated with Rapid Immunity to SARS-CoV-2) cohort to follow health care workers (HCWs) of the Mount Sinai Health System with and without documented COVID-19 over time. Full-length spike-binding IgG antibody titers were measured every 2 to 4 weeks using a sensitive and specific quantitative ELISA (13). In addition, data on potential exposures as well as clinical signs and symptoms suggestive of SARS-CoV-2 infection were collected at the same time intervals.

Here, we report our findings regarding the kinetics of SARS-CoV-2 spike-binding IgG antibody titers over time and the protection from reinfection in a high-risk work environment.

## RESULTS

**FIG 1:**
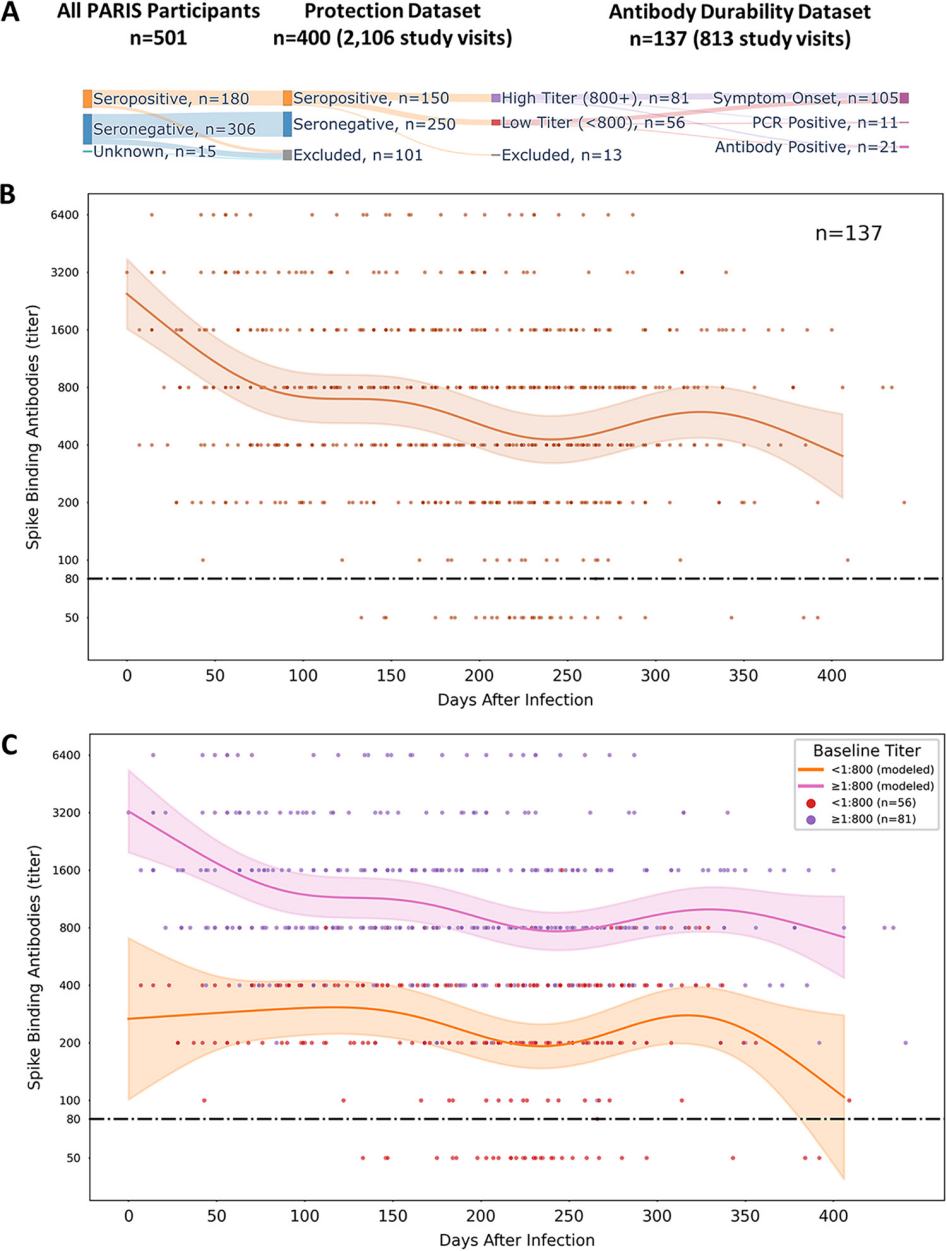
Modeling SARS-CoV-2 antibody durability in PARIS participants. (A) Overview of the PARIS cohort data sets (protection, antibody durability) selected for the analysis of humoral responses mounted upon infection. (B) Durability of SARS-CoV-2 spike-binding antibodies over time. SARS-CoV-2 IgG binding antibody dynamics after infection were described in 137 PARIS participants by an additive mixed model. The early waning period is followed by stabilization. The date of SARS-CoV-2 infection was determined by a positive nucleic acid amplification test, onset of COVID-19 symptoms, or the date of first positive antibody test. Most participants were infected in the first wave of the pandemic. The limit of detection of the SARS-CoV-2 IgG antibody ELISA is set at a titer of 1:80 (dashed black line). The brown line represents the mean antibody titer value predicted by the model, and the light brown shaded region represents the 95% confidence interval of the mean. Individual antibody values are represented by dots. (C) SARS-CoV-2 antibody durability depends on the initial levels of antibodies. The antibody durability group was split by the antibody titer at enrollment (pink, ≥1:800, *n* = 81; orange, <1:800, *n* = 56). Both groups demonstrate broadly similar dynamics, with the early waning period being most evident in the group with higher initial SARS-CoV-2 spike-binding antibody titers. Titers equal to or above 1:800 were defined as high. The pink and orange lines represent the mean antibody titer value predicted by the model, and the light pink/orange shaded regions represent the 95% confidence interval of the mean. The dashed black line represents the 1:80 cutoff value. Individual antibody values are represented by dots (≥1:800, purple; <1:800, red).

We analyzed the spike-binding IgG antibody levels of 400 PARIS participants with (*n* = 150) or without previous COVID-19 (*n* = 250) collected every 2 to 4 weeks for a median of 84 days (interquartile range [IQR], 55 to 169) from April 2020 to August 2021. The majority of participants were female (68%) with a median age of 35 years (range, 19 to 75; IQR, 30 to 45). The demographics of the cohort are summarized in Tables 1 and 2. Approximately one-third of the participants self-reported as performing high-risk tasks as part of their work assignments. Most participants with spike-binding antibodies at study enrollment (92.7%, 139/150) were infected during the first pandemic wave when NYC was one of the epicenters of the pandemic (March to May 2020). Of the remaining 11 participants, 7 were infected in the summer and fall of 2020 prior to enrolling into PARIS, and 4 participants did not recall having any symptoms suggestive of COVID-19. Two PARIS cohort data sets were used to analyze durability and effectiveness of serological responses (protection data set and antibody durability data set in Fig. 1A and Tables 1 and 2).

**TABLE 1:** Characteristics of the PARIS participants included in the protection data set^*a*^

Protection data set parameter	Total	Seronegative	Seropositive
No. (%) of participants	400 (100.0)	250 (62.5)	150 (37.5)
Sex (no. [%])			
Female	273 (68.3)	175 (70.0%)	98 (65.3%)
Male	126 (31.5)	74 (29.6%)	52 (34.7%)
Prefer not to say	1 (0.3)	1 (0.4)	0 (0.0)
Age (no. [%])			
<40	250 (62.5)	162 (64.8)	88 (58.7)
40+	149 (37.3)	87 (34.8)	62 (41.3)
Missing	1 (0.3)	1 (0.4)	0 (0.0)
Days enrolled (median, IQR)	84 (55–169.25)	84 (55.25–174)	84 (55–167)
Seroreversion (no. [%])	9 (2.3)		9 (6.0)

a Protection data set (*n* = 400). Parameter values are presented as number (%) unless indicated otherwise.

**TABLE 2:** Characteristics of the PARIS participants included in the antibody durability data set^*a*^

Antibody durability data set parameter	Total	High baseline titer (800+)	Low baseline titer (<800)
No. (%) of participants	137 (100.0)	81 (59.1)	56 (40.9)
Sex (no. [%])			
Female	89 (65.0)	59 (72.8)	30 (53.6)
Male	48 (35.0)	22 (27.2)	26 (46.4)
Age (no. [%])			
<40	80 (58.4)	37 (45.7)	43 (76.8)
40+	57 (41.6)	44 (54.3)	13 (23.2)
Days enrolled (median, IQR)	84 (56–168)	108 (56–192)	69 (55–112)
Days between SO^*b*^ and enrollment	154 (91–203)	147 (63–196)	182 (133–217)
Baseline SARS-CoV-2 antibody titer^*c*^	1:730 (2.67)	1:1,419 (1.93)	1:279 (1.52)
Seroreversion (no. [%])	8 (5.8)	0 (0.0)	8 (14.3)

a Antibody durability data set (*n* = 137). Parameter values are presented as number (%) unless indicated otherwise.

b SO, symptom onset.

c Geometric mean (standard deviation).

To model spike-binding antibody kinetics, we analyzed a total of 813 distinct spike-binding measurements from 137 participants (median, 5 study visits; IQR, 4 to 8 visits per participant, longitudinal follow-up of 2 to 6 months up to 400 days postinfection) (see Tables 1 and 2; see also Fig. S1 in the supplemental material). The date of symptom onset or positive nucleic acid amplification test (NAAT) was used as day 0 when available. Alternatively, we used the date of first positive SARS-CoV-2 antibody assay as day 0 for 21 participants. Spike-binding IgG antibody titers were highly variable among COVID-19 survivors, with titers ranging between 1:80 and 1:6,400. The majority (59.1%) of participants had SARS-CoV-2 binding antibody titers at or and above 1:800 at their baseline visit. We noted that the antibody levels decreased over the first 3 months, followed by a relative stabilization that persisted up to 1 year post-infection (Fig. 1B). Given the large variation in the initial antibody levels, we modeled whether the slopes for those with titers at or above 1:800 were different from the slopes measured for those with lower antibodies (less than 1:800). SARS-CoV-2 spike-binding antibody kinetics between the two groups were comparable, with the initial decay being more pronounced in the high antibody group. The slight “wiggliness” of the fit lines from 150 to 350 days should, however, not be attributed to a biological effect. The overlapping confidence intervals suggest a relative stability in titers during this period (Fig. 1C).

10.1128/mbio.01784-22.2 FIG S1 Duration of study follow-up for unvaccinated PARIS participants included in the protection dataset. (A) The time between first and final pre-vaccine antibody measurements for each participant is shown graphically. The total length of follow-up for unvaccinated participants varies substantially since the SARS-CoV-2 vaccine rollout for health care workers started in the middle of December 2020. (B) Enrollment in the PARIS cohort and follow-up are shown. PARIS began in April 2020 and continued recruitment throughout the year. The graph summarizes all of the pre-vaccination visits for each participant. The number of unvaccinated participants in PARIS declined sharply when SARS-CoV-2 vaccinations became available in December 2020 limiting the number of follow-up visits included in the study. Vaccination became mandatory for Mount Sinai staff members in September 2021. Download FIG S1, PDF file, 0.1 MB. Copyright © 2022 Kubale et al. 2022 Kubale et al. https://creativecommons.org/licenses/by/4.0/ This content is distributed under the terms of the Creative Commons Attribution 4.0 International license.

We next tested whether demographic variables, such as sex or age, were associated with spike antibody durability by modeling the impact of sex and age on antibody levels over the course of the observation period. We found that more advanced age (e.g., 40 years or older) was associated with 1.62-fold higher antibody levels (95% confidence interval [CI], 1.20 to 2.19) compared to those of younger participants. Sex was also associated with the level of SARS-CoV-2 spike antibodies, with antibody levels being 1.40-fold higher in female participants (95% CI, 1.03 to 1.92) than in male participants (see Table S1 in the supplemental material).

10.1128/mbio.01784-22.1 TABLE S1 Additive mixed model results regarding variables influencing the level of SARS-CoV-2 spike-binding IgG antibodies. The model indicates significant effects on SARS-CoV-2 antibody levels due to both age (>40 years of age) and sex (female). Significance was set at *P* < 0.05. Estimates are on a log 2 scale. Download Table S1, DOCX file, 0.04 MB. Copyright © 2022 Kubale et al. 2022 Kubale et al. https://creativecommons.org/licenses/by/4.0/ This content is distributed under the terms of the Creative Commons Attribution 4.0 International license.

**FIG 2:**
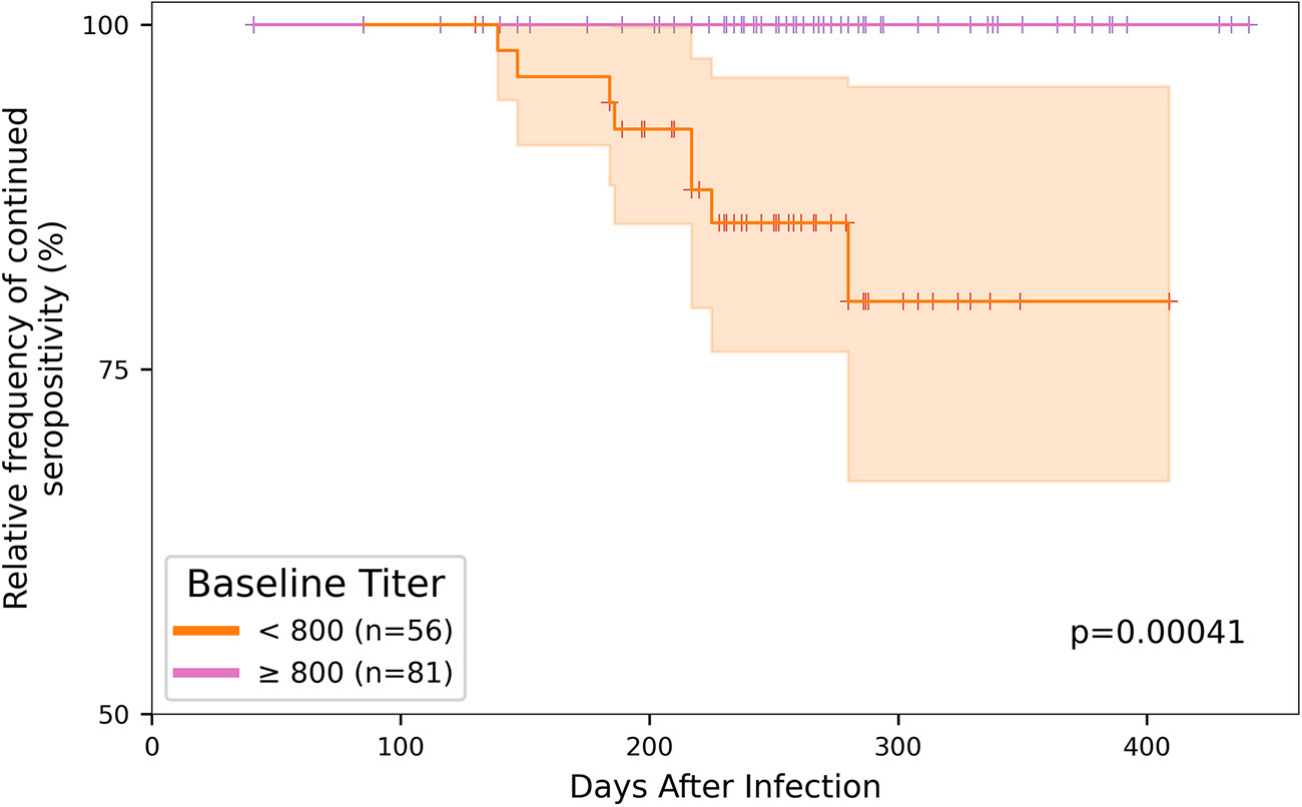
Risk of seroreversion in the antibody durability data set. The Kaplan-Meier estimate indicates that the risk of seroreversion was significantly higher for the participants with lower antibody titer at the time of study enrollment (*n *= 56 < 1:800 titer [orange]; *n* = 81 ≥ 1:800 titer [pink]). Of the 137 participants included in the antibody durability data set, only eight (6%) participants seroreverted, all of which were initially in the lower antibody titer group.

While all of the participants with documented SARS-CoV-2 infection mounted detectable antibody responses, we wondered whether seropositive individuals would turn seronegative during the observation period. We found that 6% (8/137) of the initially seropositive participants in the antibody durability data set tested negative on subsequent visits occurring over up to 11 months of study follow-up. All eight of these participants were initially in the lower baseline antibody group (below 1:800 titer), pointing to a significantly higher risk of seroreversion for individuals with initially lower antibody titers (Fig. 2, Kaplan-Meier estimate).

**FIG 3:**
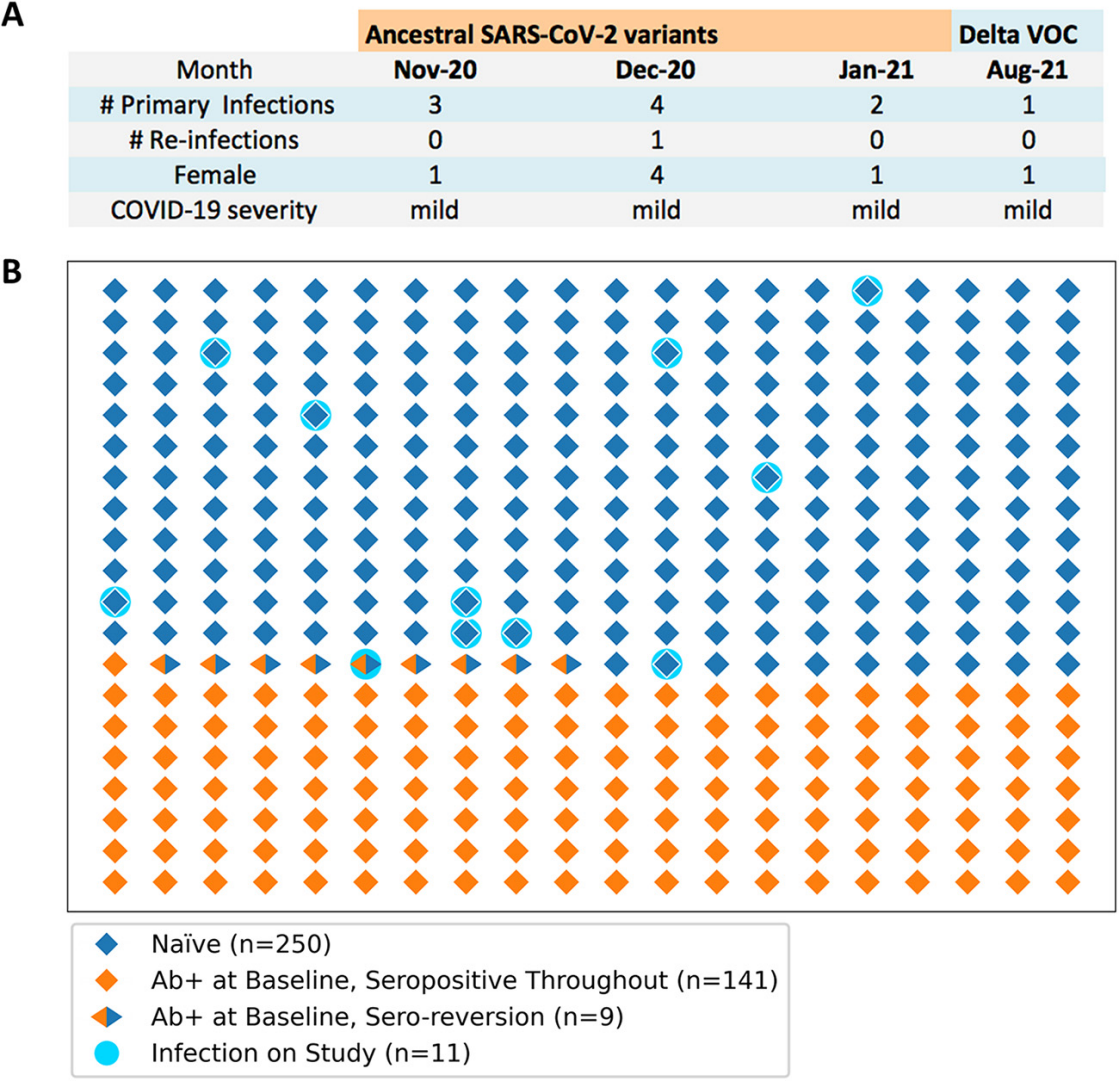
SARS-CoV-2 infections occurring in PARIS participants. (A) Summary of the new infections documented in the protection data set (*n* = 400). The circulating viral variants, the month of infection, the sex of the infected participants, and COVID-19 severity is listed for the 11 SARS-CoV-2 infections documented in the protection data set between April 2020 and August 2021. (B) Graphic representation of the frequency of SARS-CoV-2 infections in the seropositive (*n* = 149, orange diamonds) and seronegative (*n* = 251, blue diamond) study participants. Each diamond symbol represents a distinct study participant. Participants who seroreverted are indicated by orange/blue symbols. On-study infections occurred in 11 study participants without detectable SARS-CoV-2 spike-binding antibodies (turquoise circles). 10/11 participants were naive (blue diamond symbols). One participant had a documented prior SARS-CoV-2 infection but displayed no antibodies at the time of infection.

Finally, we tested whether spike-binding IgG antibodies were associated with protection from reinfection with genetically similar SARS-CoV-2 variants. Between July 2020 and August 2021, we documented a total of 11 new SARS-CoV-2 infections in PARIS participants (Fig. 3A). Of note, 10/11 of these infections occurred at a time when only ancestral viral variants circulated in the NY metropolitan area (Fig. 3A). All but one of the SARS-CoV-2 infections occurred in naive participants. One infection was found in a participant with prior COVID-19, albeit without detectable antibodies at time of reinfection (Fig. 3B). Thus, detectable spike-binding IgG antibodies mounted upon infection are associated with significant protection from reinfection (Fisher’s exact test, *P* = 0.001) in this pre-vaccine and pre-Omicron era of the COVID-19 pandemic.

## DISCUSSION

Several studies have evaluated the durability of serum SARS-CoV-2 IgG antibodies (14). While immune responses to SARS-CoV-2 infection and vaccination and their respective protective effects have been analyzed at rapid speed and in much detail, many open questions remain. We leveraged the fact that most seropositive PARIS participants were infected in the first pandemic wave (March to April 2020) with very homogenous SARS-CoV-2 strains (15). An additional strength of the PARIS cohort is the frequent, longitudinal sample and data collection (every 2 to 4 weeks), which allows for a high level of granularity in the modeling of the durability and effectiveness of SARS-CoV-2 antibody responses. We first evaluated the stability of spike-binding IgG antibody titers over time. Typical antibody responses to infection are characterized by an initial strong peak, driven by short-lived plasmablasts in the peripheral blood circulation, followed by a decline and an eventual stabilization at a level of antibody that is produced by long-lived plasma cells in the bone marrow (16–19). This was exactly the pattern that we observed in our analysis: high SARS-CoV-2 antibody titers declined initially but stabilized over the following months. Overall, seroreversion was rare, with only 6% of the COVID-19 survivors having antibody levels wane to below the level of detection of our sensitive full-length spike IG binding antibody ELISA. Interestingly, there were large differences in the spike-binding antibody titers across participants, with those 40 years and older having higher antibody titers compared to those of younger individuals. This phenomenon has been observed before (20, 21). Our observation that female participants have higher antibody titers than male participants stands in contrast to several previous studies reporting that males have higher SARS-CoV-2 antibody levels (4). The PARIS cohort comprises mostly younger and overall healthy health care workers with almost exclusively mild infections, so it is conceivable that this sex difference becomes less apparent when more severe COVID-19 manifestations, known to result in higher antibody levels (21), are included in the analysis. Of note, other than the magnitude, there was no sizable difference in the kinetics of antibody levels depending on age or sex. Additional studies in longitudinal cohorts with as frequent sampling as done in the PARIS cohort are needed to independently replicate our observations.

Several studies from the pre- (21–27) and post-Delta (B.1.617.2) (28, 29) era suggest that protection from reinfection ranges around 80 to 90% if the circulating SARS-CoV-2 variants are antigenically similar to the ones responsible for the initial infections. Only the appearance of Omicron has led to an increase of reinfections (29, 30). Our cohort study supports this notion since we did not document any reinfections in study participants who were previously infected and maintained detectable levels of spike-binding antibodies. Indeed, 10 naive individuals and one individual with an initially low titer who seroreverted prior to reinfection were infected during the observation period. These findings suggest significant protection from reinfection and hint at the importance of the level of spike-binding antibody titers in protection. Of note, the presence of spike-binding antibodies was also correlated with protection in several other studies (22, 31–33). Antibody titers against the receptor binding domain and the full-length spike, as well as neutralizing antibodies, have recently been proposed as correlates of protection of vaccine-induced immunity (34–36). Importantly, the data in these studies were generated before the Omicron variants started to circulate at larger scale in our community. Similarly, the current analysis was conducted prior to the circulation of SARS-CoV-2 variants of concern in the NYC metropolitan area. Our data underscores that spike-binding antibodies protect against infection with antigenically similar viral strains. Protection against heterologous, antigenically distinct variants, such as Omicron, is likely limited based on the pronounced reduction in virus neutralization (37–40).

This study has several strengths. First, the prospective nature of the cohort allowed us to assess how antibody responses against SARS-CoV-2 following natural infection changed over time. Second, the repeated sampling/testing of this study provides a perspective on a much more granular scale than previous analyses. Finally, this study is based on data collected prior to the introduction of SARS-CoV-2 vaccines and the wide circulation of variants of concern that are highly antigenically distinct (e.g., Omicron). As such, it provides a useful baseline against which newer data can be compared to answer important questions regarding the relative severity of new variants, the strength and durability of antibody responses against SARS-CoV-2 variants, and the impact of immune histories on the breadth of immune responses.

This analysis did, however, also have a few limitations. First, since we started enrollment during the first wave, a good portion of participants were unable to get molecular tests at the time of infection, and we relied on retrospective reports of clinical signs and symptoms suggestive of COVID-19 for illness onset date. As such, recall bias in reported illness onset is a possibility. However, we anticipate that this exerted only a minor impact on our conclusions given the relatively homogenous exposures of participants who are all health care workers. Second, with health care worker vaccination beginning in December 2020, we were unable to effectively assess how circulating variants of concern may affect one’s risk of reinfection following natural infection. The increase in vaccinated participants (excluded from this analysis), while fortunate, also resulted in a smaller sample size at the end of the follow-up period extending into August 2021.

In conclusion, our study shows that SARS-CoV-2 infection provides strong protection from reinfection, and this protection may be associated with the presence of spike-binding antibodies. In addition, it suggests that antibody levels induced by infection with ancestral SARS-CoV-2 variants are relatively stable over time and that the rate of seroreversion is low when measuring SARS-CoV-2 spike-binding IgG antibodies.

## MATERIALS AND METHODS

### Description of the PARIS cohort.

The PARIS study enrolled health care workers with and without prior SARS-CoV-2 infection to study the durability and effectiveness of the immune response to SARS-CoV-2. A total of 501 participants were enrolled between April 2020 and August 2021. The study protocol was reviewed and approved by the Mount Sinai Hospital Institutional Review Board (IRB-20-03374). All participants provided written informed consent. Samples were coded prior to processing and testing. Blood was collected at 2- to 4-week intervals regardless of the serostatus at enrollment. For this analysis, the cohort was restricted to 400 participants enrolled prior to SARS-CoV-2 vaccination with at least 4 weeks of follow-up or two study visits prior to vaccination. At the time of enrollment, 150/400 participants were seropositive for SARS-CoV-2 spike-binding antibodies while 250/400 were seronegative. Most participants had no known immunosuppressive conditions/comorbidities. We used the data from 2,106 distinct study visits from these 400 participants to evaluate risk of SARS-CoV-2 infection and seroreversion. From this data set, we selected a subset of 137 participants with known dates of COVID-19 (symptom onset, positive SARS-CoV-2 nucleic acid amplification test [NAAT], or positive SARS-CoV-2 antibody test results) and at least two pre-vaccine study visits with SARS-CoV-2 antibody measurements. The data of 813 distinct study visits from these 137 seropositive participants provide the basis for the modeling SARS-CoV-2 spike-binding IgG antibody durability.

### Identification of new SARS-CoV-2 infections in PARIS.

One of the 11 participants who were infected during the observation period was diagnosed as part of the study using viral diagnostic NAAT, while nine participants tested positive for SARS-CoV-2 outside of the Mount Sinai Health System. One asymptomatic infection was identified by seroconversion (from negative to a titer of 1:400).

### SARS-CoV-2 full-length spike-binding antibody measurements.

Antibody titers were determined using a two-step ELISA protocol (13), in which serum samples are screened at a single dilution (1:50) for IgG against the recombinant receptor binding domain (RBD) of the spike protein from SARS-CoV-2 (Wuhan-Hu-1), followed by detection of antibodies against the full-length spike protein (also Wuhan-Hu-1). End-point titers were determined by serially diluting serum (from 1:80/1:100 to 12,800). Briefly, 96-well microtiter plates (Thermo Fisher) were coated with 50 μL/well of recombinant RBD (2 μg/mL) overnight at 4°C. Plates were washed three times with phosphate-buffered saline (PBS) (Gibco) supplemented with 0.1% Tween 20 (PBS-T) (Fisher Scientific) using an automatic plate washer (BioTek). Plates were then blocked with PBS-T containing 3% milk powder (American Bio) for 1 h. Serum was heat-inactivated and serially diluted (2-fold) in PBS-T 1%-milk powder, starting at 1:50 initial dilution for RBD ELISA and at 1:80/1:100 dilution for full-length spike ELISA. Samples were added to the plates and incubated for 2 h. Plates were washed three times with PBS-T, and 50 μL/well of anti-human IgG (Fab-specific) horseradish peroxidase antibody (produced in goat; Sigma; A0293) diluted to 1:3,000 in PBS-T, 1% milk powder, were added to each well. After 1 h incubation at room temperature, plates were washed three times with PBS-T, and 100 μL/well of SigmaFast *o*-phenylenediamine dihydrochloride (Sigma) was added for 10 min, followed by addition of 50 μL/well of 3 M hydrochloric acid (Thermo Fisher) to stop the reaction. Optical density was measured at a wavelength of 490 nm using a plate reader (BioTek). Endpoint titers, expressed as the last dilution before the signal dropped below an optical density at 490 nm (OD_490_) of 0.15, were calculated in excel, and data were plotted using GraphPad Prism 9. True-positive samples were defined as samples that exceeded an OD_490_ value of 0.15 at a serum dilution of 1:80 (11).

### Assessment and modeling of SARS-CoV-2 spike-binding antibody durability over time.

To assess how spike antibody titers changed over time, we fit an additive mixed model using the mgcv package (version 1.8-36) for R (version 4.1.1). Participants were excluded from the model if they never developed a detectable titer (at least 1:80) during follow-up or if the data regarding when they were infected (illness onset, NAAT positive, or antibody positive date) was missing. Day 0 was defined as the reported symptom onset date or the date of positive SARS-CoV-2 diagnostic test.

Spike titers (ranging from 1:80 to 1:6,400) were transformed to the log_2_ scale so that a 1-unit increase corresponded to a doubling of antibody titer. Sex (female, male), age (<40 years, 40+ years), and baseline titer (<1:800, ≥1:800) were included as covariates in the model, along with a random intercept for participant ID to account for repeated measures. Finally, a penalized spline term was included to model antibody titer over time. We fit this model in the following two ways: (i) assuming that antibody decay occurred at the same rate over time regardless of baseline titer, and (ii) allowing antibody decay over time to vary by baseline titer. In the second model, a distinct smoothing function was fit for each baseline titer group. Data is available upon request, and the code used for modeling is available on GitHub at https://github.com/jkubale/paris.

### Determination of the frequency of spike-binding antibody seroreversion.

Seropositive participants who initially had measurable spike-binding antibodies but subsequently had spike-binding antibody levels below the limit of detection (1:80) on two consecutive visits were defined as having seroreverted. We examined the probability of seroreversion over time for those with low (<1:800) and high (≥1:800) baseline titers by calculating the probability of survival (not seroreverting) via the Kaplan-Meier estimator.

### Assessment of protection against reinfection.

We explored whether participants with a detectable antibody titer had a lower probability of incident SARS-CoV-2 infection. New SARS-CoV-2 infections were identified by positive NAAT or by SARS-CoV-2 antibody seroconversion. The participant immune status was based on the most recent spike-binding antibody titer preceding the infection. Participants with detectable spike-binding antibody titers were compared to those without detectable titers using Fisher’s Exact Test. All analyses were performed using R version 4.1.1. All figures were rendered using seaborn version 0.11.1, matplotlib version 3.3.4, and Python version 3.9.5.
