# Supplementary material for: SARS-CoV-2 Spike-Binding Antibody Longevity and Protection from Reinfection with Antigenically Similar SARS-CoV-2 Variants
Source: mBio. 2022 Aug 23;13(5):e01784-22. doi: 10.1128/mbio.01784-22 (PMC9600418; doi:10.1128/mbio.01784-22)
Supplement: FIG S1 [file mbio.01784-22-s0001.pdf]

Supplemental Appendix

SARS-CoV-2 spike-binding antibody longevity and protection from re-infection with antigenically similar SARS-CoV-2 variants

John Kubale[1#], Charles Gleason[2,6#], Juan Manuel Carreño[2,6], Komal Srivastava[2,6], Gagandeep Singh[2,6], PARIS Study Team[2,6], Aubree Gordon[1*], Florian Krammer[2,5,6*], Viviana Simon[2,3,4,5,6*]

Supplemental Figure S1

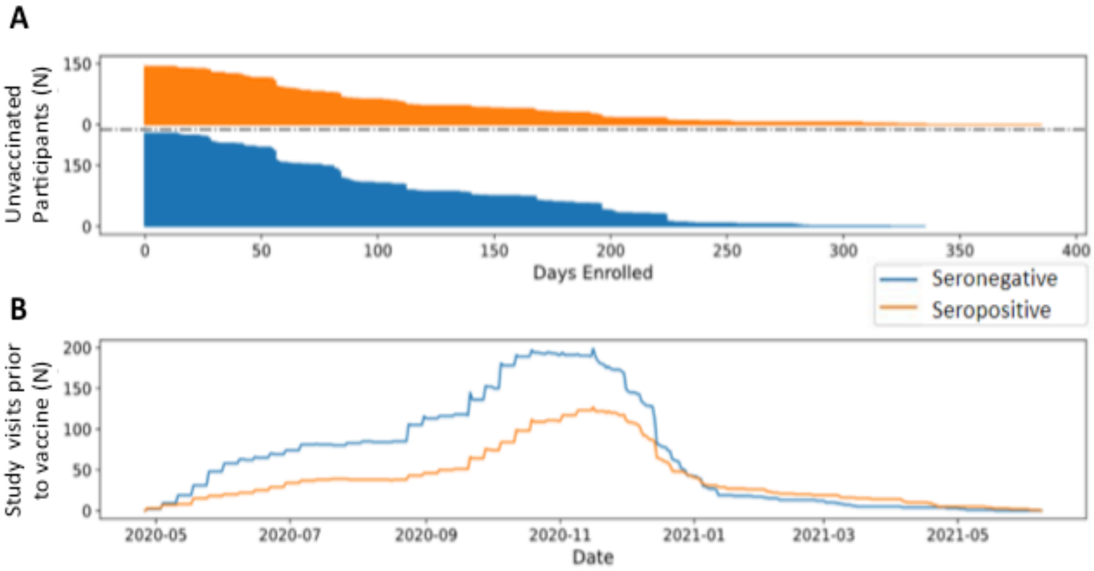

Supplemental Figure S1: Duration of study follow-up prior for unvaccinated PARIS participants included in the protection dataset

(A) The time between first and final pre-vaccine antibody measurements for each participant is shown graphically. The total length of follow-up for unvaccinated participants varies substantially since the SARS-CoV-2 vaccine rollout for health care workers started in the middle ofDecember 2020.

(B) Enrollment in the PARIS cohort and follow up are shown. PARIS began in April 2020 and continued recruitment throughout the year. The graph summarizes all the pre-vaccination visits for each participant. The number of unvaccinated participants in PARIS declined sharply when SARS-CoV-2 vaccinations became available in December 2020 limiting the number of follow up visits included in the study. Vaccination became mandatory for Mount Sinai staff members in September 2021.
