# Supplementary material for: SARS-CoV-2 Spike-Binding Antibody Longevity and Protection from Reinfection with Antigenically Similar SARS-CoV-2 Variants
Source: mBio. 2022 Aug 23;13(5):e01784-22. doi: 10.1128/mbio.01784-22 (PMC9600418; doi:10.1128/mbio.01784-22)
Supplement: TABLE S1 [file mbio.01784-22-s0002.docx]

**Supplemental Appendix**

**SARS-CoV-2 spike-binding antibody longevity and protection from re-infection with antigenically similar SARS-CoV-2 variants**

John Kubale^1#^, Charles Gleason^2,6#^, Juan Manuel Carreño^2,6^, Komal Srivastava^2,6^, Gagandeep Singh^2,6^, PARIS Study Team^2,6^, Aubree Gordon^1*^, Florian Krammer^2,5,6*^, Viviana Simon^2,3,4,5,6*^

**Supplemental Table S1**: **Additive mixed model results regarding variables influencing the level of SARS-CoV-2 spike binding IgG antibodies.**

The model indicates significant effects on SARS-CoV-2 antibody levels due to both age (>40 years of age) and sex (female). Significance was set at p < 0.05. Estimates are on a log 2 scale.

|  | **Estimate** | **Std. Error** | **t value** | **p value** |
| --- | --- | --- | --- | --- |
| (Intercept) | 8.5592 | 0.2171 | 39.434 | < 2e-16 *** |
| Sex (Female) | 0.4868 | 0.2299 | 2.118 | 0.03453 * |
| Age (>40 yrs) | 0.6983 | 0.2210 | 3.159 | 0.00165 ** |
